# Advances and challenges of immunocheckpoint inhibitors in the treatment of primary liver cancer

**DOI:** 10.3389/fgene.2022.1005658

**Published:** 2022-09-30

**Authors:** Meng Hu, Weirong Yao, Qinglin Shen

**Affiliations:** ^1^ Department of Oncology, Jiangxi Provincial People’s Hospital, the First Affiliated Hospital of Nanchang Medical College, Nanchang, China; ^2^ Institute of Clinical Medicine, Jiangxi Provincial People’s Hospital, the First Affiliated Hospital of Nanchang Medical College, Nanchang, China

**Keywords:** primary liver cancer, immune checkpoint inhibitors, immunotherapy, advances, challenges

## Abstract

Primary liver cancer (PLC) is one of the most common malignant tumors, which clinically characterized by occult onset, rapid development, easy recurrence and poor prognosis. With the rapid development of tumor immunotherapy research, tumor immunotherapy has also achieved remarkable clinical efficacy, and jointly promoted the overall improvement of tumor immunology from mechanism research to clinical transformation, from single discipline to multi-disciplinary integration. Immunotherapy has obvious advantages in treatment-related toxicity and efficacy compared with traditional therapy. In hepatocellular carcinoma (HCC), immunotherapy alone or in combination with other therapies may help to control tumor progression, and there are many immune checkpoint inhibitors (ICIs) widely used in clinical or ongoing clinical trials. However, tumor immunology research is still facing many challenges. How to effectively evaluate the efficacy, whether there are related biomarkers, the generation of immune tolerance and the lack of clinical trials to objectively evaluate the efficacy are still urgent problems to be solved, but it also brings new research opportunities for basic and clinical immunology researchers. The study of treatment of ICIs of PLC has become a hot spot in clinical research field. This paper summarizes and prospects the research progress and challenges of ICIs for PLC.

## Introduction

Primary liver cancer (PLC) includes hepatocellular carcinoma (HCC), cholangiocarcinoma (CCA) and mixed hepatocellular carcinoma -cholangiocarcinoma (HCC-CCA). HCC accounts for around 90% of the total number of PLC, with incidence rate of fifth in men and eighth in women. According to the global cancer statistics in 2020, HCC is the sixth largest cancer in the world, with the death rate ranking the fourth in the world (Sung et al., 2021). There are at least 700,000 new cases of HCC raised every year. The number of patients with HCC in China accounts for about 50–55% of the total number of patients in the world (Jemal et al., 2013), and the mortality accounts for 50% of the total number of patients with HCC in the world. The main pathogenic factors of HCC include: hepatitis B virus (HBV) infection, alcoholic liver disease, diabetes, and some metabolic diseases (Mcglynn et al., 2015). The routine treatment of HCC consists of surgery, chemoradiotherapy, targeted therapy and immunotherapy (De Lorenzo et al., 2018; Tai et al., 2019). Base of the characteristics of PLC and the degree of malignancy, most of the patients with PLC are in the middle and advanced stage when they are initially diagnosed, who would almost lose the chance of surgery treatment, so, the systemic drug therapy is considered as the proper management (Waese et al., 2017; Rizzo et al., 2021b). In December of 2005, the food and Drug Administration (FDA) approved Solafeni for the treatment of first-stage renal cancer. Since that, the period of targeted treatment of PLC was officially initiated, then, the molecular targeted drugs such as Lenvatinib and Regorafenib were licensed subsequently in treatment of HCC. However, the curative effect of tyrosine kinase inhibitors (TKIs) is limited due to the emergence of drug resistance (Saffo and Taddei, 2019; Rizzo et al., 2020).

With the rapid development of molecular biology, studies have found that the liver is important immune organ of the body (Solter and Philip, 2005; Szabo et al., 2018), because there are a large number of macrophages and immune cells in the liver microenvironment, which makes it form a very complex immune tolerance microenvironment (Peterson., 2012). Therefore, immunotherapy for PLC arises at the historic moment. Studies have confirmed that immunotherapy could enhance the body’s immune response, break the immune tolerance, activate the body’s immune cells to recognize and kill tumor cells, so as to obtain anti-tumor effect (Scheinberg and Pinilla-Ibarz, 2006; Scheinberg and Pinilla-Ibarz, 2009). Immunotherapy for PLC would stimulate the body’s immune response to tumor cells and regulate the immune microenvironment of PLC through various ways of immune activation, so as to achieve the anti-tumor effect through the interaction of immune cells and molecules (Cao et al., 2005; Cantor et al., 2013; Rizzo et al., 2021a). Although the immunotherapy of PLC has made gratifying progress, it still faces many problems, e.g., the related immune escape and combined therapy of PLC. This paper here focuses on the summarization of the advances and challenges of ICIs in the treatment of PLC.

## Immunosuppression mechanism in microenvironment of PLC

First of all, tumor microenvironment (TME) mainly refers to the internal and external environment of the body where tumor occurs and develops, which plays an important role in the process of tumor development, immune escape, body immunosuppression and anti-tumor (Chen and Hua, 2012). Under physiological conditions, intestinal metabolites and bacteria would enter the liver through the portal system, and act as antigens to stimulate the liver immune system to maintain homeostasis and avoid excessive autoimmune reaction, which is feasible to establish immune tolerance microenvironment. Therefore, PLC is also called immune amnesty organ (Fernández et al., 2019).

China is a traditional country with a large population of hepatitis B. PLC is often derived from chronic hepatitis B cirrhosis and chronic hepatitis patients. Due to the interaction of various inflammatory cells, the liver is immersed in more complex chronic inflammatory microenvironment (Yoon et al., 2010; Altomonte and Ebert, 2014). With the development of molecular biology and the studies in mechanism of malignant tumor, the hepatoma cells are identified in a highly inhibited immune microenvironment. Through down regulation of the main histocompatibility complex-I(MHC-I), secretion of immunosuppressive cytokines and negative co-stimulation signals (Lowe et al., 2014), the host immunosuppression is induced, result in avoiding the autoimmune response. The particularity of PLC leads to the complexity and challenge of its immunotherapy (Yuan et al., 2017; Nguyen et al., 2021). The results show that (Shiraha et al., 2020), tumor related fibroblasts in TME can release a large number of immunosuppressive related molecules, such as prostaglandin E2; In addition, the risk of recurrence of PLC after liver transplantation is correlated with Th1 cells and interferon-γ, and the high expression of these cytokines may be related to the prognosis of tumor. In addition, CD8^+^ T cell is one of the main immune cells that able to identify and kill tumor cells. In the microenvironment of PLC, the function of CD8^+^ T cell is inhibited to promote the rapid growth of HCC cells (Du and Wang, 2011). For example, in PLC, bone marrow cells differentiate into more immature myeloid cells, which can affect the immune microenvironment of PLC at a certain stage (Perussia et al., 1984). This cell is also called the marrow derived suppressor cells (MDSCs) due to its immature characteristics and remarkable diversity. Different main immune cells were differentiated in variety of environments and time, including: dendritic cells (DCs), macrophages and neutrophils, which are called tumor related DCs, tumor associated macrophages (TAMs) (Thiem et al., 2021), tumor associated neutrophils (TANs). The microenvironment of tumor also includes cancer associated fibroblasts (CAFs), tumor infiltrating lymphocytes (TILs). Simultaneously, there are many immunosuppressive pathways in the microenvironment of PLC, which exert the progress of PLC and immune escape (Zhu et al., 2019). For instance, in the process of chronic hepatitis caused by long-term hepatitis virus (mainly hepatitis B), many kinds of inhibitory immune factors would be secreted, which promote the occurrence and proliferation of malignant tumor cells (Timperi and Barnaba, 2020); The endogenous cell cycle related kinase (CCRK) can be applied into the liver through EZH2/NF-kB signaling pathway to upregulates the production of IL-6, inducing MDSCs to gather in TME, which plays a role in stimulating tumor growth (Zhou et al., 2017); Some studies have shown that the proliferation of liver tumor cells will generate local hypoxia to induce the increase of MDSCs, leading to the progress of tumor cell cloning (Chiu et al., 2017). In addition, there are also studies release TANs could raise macrophages and Tregs cells into hepatoma cells, and enhance the growth and progress of HCC (Zhou et al., 2016; Jiang et al., 2017). These immunosuppressive factors play a regulatory role in the occurrence and development of PLC to different degrees.

In short, the occurrence and development of PLC need to be formed through a variety of ways and factors, and the mechanism is relatively complex.

## Current status of ICIs for HCC

At present, the studies of immune checkpoint inhibitors (ICIs) is hot in the research of malignant tumor, especially in the immunotherapy of lung cancer, and then spread into the research of a variety of other malignant tumors. The targets of immunosuppressive agents mainly include programmed death-1 (PD-1)/programmed death ligand 1 (PD-L 1) and cytotoxic T lymphocyte associated antigen-4 (CTLA-4), also many other encouraging clinical results have been reported. Progress of various ICIs in the treatment of PLC summarized in Table 1.

**TABLE 1:** Progress of various immune checkpoint inhibitors in the treatment of primary liver cancer.

Drug name	Characteristics of cohort study	Target (mechanism)	Patient selection	Intervention measures	grouping	Clinical results (ORR, PFS, OS)	Main side effects
Atilizumab + Bevacizumab (A+ T)	Imbravei50 phase III multicenter study	PD-1+ VEGF inhibitor	501 unresectable HCC patients who had not received systematic treatment before	They were randomly assigned into the experimental group and the control group according to 2:1. The experimental group received 1200 mg intravenous infusion of atilizumab, followed by 15 mg/kg intravenous infusion of bevacizumab on the same day, Q3W; The control group was treated with sorafenib 400 mg orally twice a day until the disease progressed or intolerable toxicity appeared	A+ T *vs*. Sorafenib	A+ T *vs.* Sorafenib: mOS 19.2 *vs*. 13.4 m(HR = 0.66, *p* = 0.0009), mPPS 6.9 *vs*. 4.3 m,	38% of people had serious AE (Grade 3–4), and the most common AEs (in ≥20% of patients) were hypertension, fatigue and proteinuria
						ORR 29.8% *vs*. 11.3%	
Camrelizumab + apatinib	Non-random, open, multi center, phase II project carried out by 25 centers in China	PD-1+VEGFR-2 inhibitor	Phase II clinical study on the first-line and second-line treatment of 190 cases of advanced liver cancer in rescue	Camrelizumab (intravenous, 200 mg [for body weight ≥50 kg] or 3 mg/kg [for body weight <50 kg], Q2W) + Apatinib (250 mg/day, Q4W)	Camrelizumab + Apatinib *vs*. Apatinib	In the first-line treatment group, mOS was 20.3 m, mPFS was 5.7 m, ORR was 34.3%	The frequency of TRAs above Grade 3 was 78.6%
Nivolumab + Ipilimumab (O+ Y)	Checkmate 040 cohort 4 phase I/II global multicenter single arm study	PD-L1+CTLA-4	Second line phase I/II study of 148 cases of advanced liver cancer	The patients were randomly assigned according to the ratio of 1 ∶ 1 ∶ 1. They received 1 mg/kg of Nivolumab combined with 3 mg/kg of Ipilimumab Q3W (4 doses), and then 240 mg of Nivolumab Q2W (group A); Nivolumab 3 mg/kg combined with Ipitumab 1 mg/kg Q3W (4 doses), and then 240 mg Nivolumab Q2W (group B); Or 3 mg/kg of Nivolumab Q2W and 1 mg/kg of Ipilizumab Q6W (Group C)	Group A (Nivolumab 1 mg/kg, Ipilimumab 3 mg/kg, Q3W, followed by Nivolumab 240 mg Q2W after 4 courses of treatment); Group B (Nivolumab 3 mg/kg, Ipilimumab 1 mg/kg, Q3W, followed by Nivolumab 240 mg every 2 weeks after 4 courses of treatment); Group C (Nivolumab 3 mg/kg, Ipilimumab 1 mg/kg, Q6W)	(mOS : 22.8 *vs*. 12.5 m *vs*. 12.7 m, ORR: 32% *vs*. 27% *vs*. 29%)	The TRAEs of double immunosuppressants were slightly higher, 3/4 of the patients in group A had AEs, but they were controllable as a whole
Durvalumab + Tremelimumab(T + D)	Study22 VII global multicenter research	PD-L1+CTLA-4	Phase II clinical study on second-line treatment of 322 cases of advanced HCC	T300 + D75 (T 300 mg + D 1500 mg, sequential D 1500 mg after one course of treatment, Q4W)	T300 + D75 (T 300 mg + D 1500mg, sequential D 1500 mg after one course of treatment, Q4W)	mOS: 18.7 *vs*. 11.7 m *vs*. 17.1 *vs*. 11.3 m; ORR: 22.7% *vs*. 9.6% *vs*. 7.2%*vs*. 9.5%	Grade 3–4 AE: 35.1% *vs*. 17.8%*vs*.
				D 104 (D 1500 mg Q4W);	T69 (T 750mg, Q4W in the first 7 cycles and Q12W after 7 cycles);		42.0% *vs*. 24.4%
				T69 (T 750mg, Q4W in the first 7 cycles and Q12W after 7 cycles)	T75 + D 84 (T 75 mg + D 1500mg, sequential D drug 1500 mg after 4 cycles, Q4W)		
				D 104 (D 1500 mg Q4W);			
Nivolumab (O)	Checkmate 459 phase III global multicenter study	PD-1	493 cases of advanced HCC	Checkmate 459 is a randomized, multicenter, phase III clinical study involving 743 patients with advanced liver cancer aged 18 or over who did not receive systematic treatment. 1: 1 after randomization, 371 patients received intravenous 240 mg navulizumab Q2W, and 372 patients took 400 mg sorafenib orally twice a day	Navuliumab(371) *vs*. Sorafenib(372)	OS:16.4 *vs*. 14.7 m; PFS:3.7 *vs*. 3.8 m; ORR: 15 *vs*. 7%	Grade 3–4 AE: 22 vs. 49%
Pembrolizumab	Keynote 240 phase III global multicenter study	PD-1	Second line treatment of 413 cases of advanced HCC	The patients were randomly assigned to receive Pembrolizumab 200 mg + best supportive treatment vs. Placebo + best supportive treatment according to 2:1, Q3W	Pembrolizumab(278)vs. Placebo(135)	OS:13.9 *vs*. 10.6 m; PFS: 3.3 *vs*. 2.8 m; ORR:18.3 vs. 4.4%	Grade 3–4 AE: 18.6vs. 7.5%
Camrelizumab	Phase II China multicenter single arm study	PD-1	220 patients were included, of which the proportion of HBV infection was as high as 83.4%	Camrelizumab 3 mg/kg, Group(Q2W)*vs*. Group(Q3W) = 110:110	Q2W(3 mg/kG)vs. Q3W(3 mg/kG)	OS:14.2 vs. 13.2 m; PFS:2.3 *vs*. 2.0m; ORR: 11.9 *vs*. 17.6%	Grade 3–4 AEs: 22%, mainly reactive skin capillary hyperplasia, and most patients mainly have grade 1–2 AEs
Sintilimab + Bevacizumab	Randomized, controlled, open phase III clinical study	Domestic PD-1 + VEGF inhibitor	571 cases of unresectable or metastatic HCC treated with first-line therapy	The patients were randomly divided into groups according to 2:1 and received Sintilimab + Bevacizumab *vs*. Sorafenib	Sintilimab + Bevacizumab *vs*. Sorafenib	mOS:Not reached(NR)*vs*.10.4m; mPFS: 4.6 vs. 2.8m; the risk of death and the risk of disease progression were reduced 43%and 44%, respectively	The incidence of grade 3–4 AEs was similar to that of sorafenib
Toripalimab + Lefatini	Single arm phase II	RTK inhibitor + PD-1 + HIC	36 adult patients with advanced HCC (≥18 years old) had ECoG score of 0–2 and child Pugh class a liver function	Lefatini (8 mg when body weight <60 kg, 12 mg when body weight ≥60kg, oral once a day) was used 3–7 days before the initial HAIC to determine tolerance. They were then treated with lenvatinib for 21 days (one cycle from day 1 to day 21), treprizumab (intravenous infusion of 240 mg on day 1), HAIC (day 1 to day 2) and FOLFOX regimen (oxaliplatin 85 mg/m2, folic acid 400 mg/m2, 5-fluorouracil 400 mg/m2 on day 1 and 5-fluorouracil 2400 mg/m2 for 224 h), Until the disease progresses or intolerable toxicity occurs	Toripalimab + Lefatini *vs*. Lefatini	PFS: 11.1 *vs*. 5.1 m, OS: Not reached(NR) *vs.* 11.0m, ORR:66.7% (CR14.1%),DCR:90.1%	Grade 3–4 TRAs (trae) occurred in 72.2% of patients. The most common were thrombocytopenia (13.9%), elevated aspartate aminotransferase (AST; 13.9%) and hypertension (11.1%)

### Current status of PD - 1/PD—L 1 blocking therapy

Ishida and his colleague (Ishida et al., 1992; Dong et al., 1999; Fitzsimmons and Sadkowsky, 2002) first discovered that PD-1 (CD279) can induce apoptosis in mouse T-cell hybridoma since 1992, there have been more and more studies on PD-1/PD-L1, which has become a “superstar” in cancer research. Among them, PD-1 is a negative costimulatory molecule of CD28 immunoglobulin superfamily of transmembrane receptor. It is a powerful inhibitor of effector T cells response. It is found in a variety of immune cells, such as T cells, B cells and NK cells. It is mainly expressed in CD8^+^ T cells. It can also be expressed in bone marrow-derived suppressor cells and Treg cells, PD-1 has two kinds of cell membrane protein ligands: PD-L1 (B7-H1/CD274) and PD-L2 (B7-DC/CD273). The process of interaction between PD-1 and PD-L1/PD-L2 is mainly that PD-1 binds with PD-L1/PD-L2, transmits the co inhibitory signal of T cells antigen receptor and inhibits the production of various cytokines by suppressing the activation of T cells, thus assisting tumor immune escape. The higher PD-1 expression level affects the proliferation and differentiation of Treg cells, and further regulates the peripheral immune tolerance response (Jilaveanu et al., 2014).

#### PD-1 inhibitors

##### Nivolumab

Nivolumab (BMS936558, MDX-1106, Opdivo) is a completely humanized IgG4 monoclonal antibody targeting PD-1. Since 2014, Nivolumab has been approved by FDA for secondary treatment of metastatic melanoma and NSCLC, and it has been approved by FDA for bladder cancer in February, 2017 and second line treatment of HCC in late stage treated by Sorafenib in September, 2017. The famous Checkmate- 040 (El-Khoueiry et al., 2017) includes two parts: phase I dose climbing study (*n* = 48) and ll phase queue extension study (*n* = 214). The results show that the disease control rate (DCR) is 58%, objective response rate (ORR) is 15%, and the overall survival (OS) is 15 months, The OS of 6 and 9 months was 66%. The ORR was 20%, DCR was 64%, and the OS in 6 and 9 months were 83 and 74%, respectively. Compared with the first-line treatment of advanced HCC Checkmate-459 (NCT02576509) (Yau et al., 2019b), the results of the global and multicenter III trials of Sorafenib showed that the median overall survival (mOS) of Nivolumab group and Sorafenib group were 16.4 and 14.7 months (*p* = 0.0419), respectively. However, the expression of PD-L1, the efficacy of Nivolumab was consistent, and the median progression free survival (mPFS) of both groups were 3.7 and 3.8 months, respectively, there was no significant difference in mPFS, with the ORR of 15 and 7%, respectively. At the same time, the safety of Nivolumab was higher, and 22 and 49% of treatment related adverse reactions (TRAEs) in the Nivolumab group and Sorafenib group were respectively.

##### Pembrolizumab

Pembrolizumab is a highly selective and humanized IgG 4 monoclonal antibody, which can target to inhibit negative regulation of PD-L signal. The clinical efficacy and safety are similar to that of Nivolumab. In 2018, a non-randomized, multicenter, open phase II single arm clinical study Keynote-224 assessed the safety and efficacy of pembrolizumab in the late stage of HCC. A total of 104 patients with advanced Child-Pugh A who were treated with Sorafenib were enrolled in the study. The study included 104 patients with advanced Child-Pugh A who had been treated with Sorafenib for 2 years, until the disease progresses or an intolerable toxic reaction occurs. The results showed that the DCR was 62%, objective remission rate was 17%, complete remission rate was 1%, mPFS reached 4.9 months, OS was 12.9 months, 12 months OS rate was 54%, the incidence of level 3 related adverse reactions was 16%, and the adverse reactions above grade 4 did not occur, and the adverse reactions were mainly caused by the increase of aspartate transaminase. It is precisely because of the Keynote-224 (Zhu et al., 2018) research results that in 2018, the FDA approved pembrolizumab for the second-line treatment of advanced HCC patients, and the second PD-1 inhibitor approved by FDA for advanced HCC. In 2019, a randomized controlled phase III clinical trial Keynote-240 (NCT02702401) compared the best support therapy with pembrolizumab for the second-line treatment of advanced HCC. The results showed that pembrolizumab had significant effect. The study included 413 patients with advanced PLC, randomly assigned to the pembrolizumab group and the control group according to 2:1, and followed up for 13.8 months. The total survival time of pembrolizumab group was prolonged for 3 months (13.9 *vs*. 10.6 m); The results showed that PFS was improved (*p* = 0.0022) in one side (*p* = 0.0238). Unfortunately, the difference did not reach the established statistical level. The ORR of pembrolizumab and placebo group was 18.3 and 4.4%, respectively. The efficacy of pembrolizumab group was long-lasting, and the median DOR was 13.8 months. In terms of safety, the treatment group had increased transaminase, bilirubin, diarrhea, rash, *etc.* most of the adverse reactions were 1-2, and 3-4 were rare. Keynote-240 study did not reach the expected results. The reasons for the end point, a value and *p* value adjustment of the study were found and the clinical research continued to be carried out. The Keynote-394 study, as a second-line treatment, is expected to be used in the randomized, double-blind, international multicenter phase III clinical study in patients with advanced Asian HCC. We believe that the clinical research of PD-1 inhibitor in the treatment of HCC will be more and more profound in the future, and that better clinical data will be obtained.

##### Camrelizumab

Camrelizumab is the first PD-1 inhibitor independently developed by HENGRUI company of China to be approved as an indication for advanced HCC. In 2019, Professor Qin Shukui led a multicenter, phase II clinical study (Qin et al., 2020) (NCT02989922) on second-line and above treatment of advanced HCC in China. A total of 220 patients were recruited in the study, of which the proportion of HBV infection was as high as 83.4%. They were randomly assigned to Camrelizumab 3 mg/kg, every 2 weeks (Q2W) and 3 mg/kg, Q3W at a ratio of 1:1. The results showed that the overall mPFS was 2.1 months, so the ORR of patients was 14.7% (the ORR of 2W group and 3W group were 11.9 and 17.6%, respectively). The OS of all patients was 13.8 months, the OS of 2W treatment group was 14.2 months, and the OS of 3W group was 13.2 months. The OS rates of all patients at 6 and 12 months were 74.7 and 55.9%, respectively. There was no significant difference in ORR between every 2 weeks and every 3 weeks. The main adverse reactions were reactive cutaneous capillary hyperplasia, and most of the patients had grade 1–2 adverse reactions. The safety was similar to that of Nivolumab and pembrolizumab. In March 2020, it was approved by the National Medical Products Administration (NMPA) as the second-line treatment for advanced HCC.

##### Tislelizumab

In the clinical research of immunotherapy for advanced HCC, immunotherapy has been unanimously recommended by domestic and foreign HCC guidelines. However, some clinical studies still fail to obtain gratifying results, such as Keynote-240 and Checkmate-459 (Vogel et al., 2020) studies, which did not reach the preset statistical significance. Tislelizumab is a PD-1 monoclonal antibody independently developed in China (Lee and Keam, 2020). It is also a humanized IgG4 anti-PD-1 monoclonal antibody. FC segment structure optimization of Tislelizumab can effectively avoid antibody-dependent cellular phagocytosis (ADCP) effect. It has a good T cell activation effect in PLC cells with immune cell aggregation, and has high affinity and specific binding ability with PD-1. FC segment of TisleliTzumab can also be optimized by genetic engineering technology to make it interact with macrophage FCɤ (Zhang et al., 2018) , On 26 December 2019, NMPA approved the market.

The BGB-A317-001 study (Desai et al., 2016; Wu et al., 2019) explored the efficacy, safety and tolerability of Tislelizumab. The study is divided into phase 1A dose climbing and dose exploration. In conclusion, after receiving Tislelizumab monotherapy for more than 12 months, patients can still maintain good tolerance. Regardless of the expression of PD-L1, Tislelizumab monotherapy can produce lasting anti-tumor effect in a variety of solid tumor patients. Currently, BGB-A317-208, BGB-A317-301 and other similar studies have been carried out.

##### Sintilimab

Sintilimab injection is a monoclonal antibody against human IgG4, which can specifically bind to PD-1 molecule on the surface of T cells, thus blocking PD-1/PD-L1 pathway leading to tumor immune tolerance, starting T cells to kill tumor cells, so as to achieve the purpose of anti-tumor. ESMO conference in 2020 reported a single arm phase II clinical study of Sintilimab combined with Anlotinib in the first-line treatment of patients with advanced HCC (Chen X. et al., 2020). In this study, a total of 14 evaluable patients had ORR as high as 42.9% (RECIST1.1), 5 patients had partial remission (PR), 1 patient had complete remission (CR), and the DCR rate was 92.9%, with good tolerance. Another clinical study, a randomized, controlled, and open phase III clinical study (Ren et al., 2020) (ORIENT-32), explored the comparison between Sintilimab combined with bevacizumab and sorafenib in the first-line treatment of unresectable HCC patients. Nearly 600 Chinese patients were included in the study. The study showed that the ORR of Sintilimab combined with bevacizumab was 5 times that of sorafenib group. The combined group reduced the risk of death and disease progression by 43%. The mOS of the two groups were not reached and 10.4 months, and the mPFS were 4.6 and 2.8 months, respectively. The safety was impressive. The incidence of grade 3–4 TRAE was only 33.7%. Because of the success of this pioneering study, it has become the first phase III clinical study of PD-1 combination therapy for first-line advanced HCC with positive results in the world. At the same time, this research result makes Sintilimab combined with bevacizumab first-line recommended by NCCN guidelines for patients with advanced HCC. Therefore, new schemes and ideas are added for patients with advanced HCC.

##### Toripalimab

On 17 December 2018, the State Drug Administration approved the first homemade PD-1 monoclonal antibody-toripalimab injection. Toripalimab is an IgG4 type monoclonal antibody with independent intellectual property rights developed by JUNSHI (Jiao Y. et al., 2020), which has a unique binding site. At the same time, a proline (S228P) point mutation was introduced into the serine protein site 228 in the hinge region of the heavy chain to increase the stability of the antibody. It has dual antitumor effects. Its anti-tumor mechanism is mainly to block PD-1/PD-L1 signaling pathway, improve T cell response activity *in vitro* and *in vivo*, and achieve T cell proliferation and anti-tumor effect (Greenwald, 2008); In addition, it mediates the endocytosis of PD-1, reduces the expression of PD-1 membrane, and relieves T cell immunosuppression. A real-world clinical study of domestic PD-1 inhibitor monotherapy for HBV related PLC (Chen J. et al., 2020) showed that the ORR of Toripalimab was 15.4%, the DCR was 53.8%, the total ORR was 17.3%, and the DCR was 72.0%. On 20 March 2021, the second CSCO-JUNSHI biological tumor immunity summit forum announced the results of initial analysis of phase II study of first-line treatment of advanced HCC with Toripalimab combined with bevacizumab, and announced the launch of international multi-center phase III clinical study (JUPITER-10, NCT04723004), A total of 54 patients with unresectable locally advanced or metastatic HCC were enrolled in this phase II study. 87% of the patients had chronic hepatitis B and more than half had extrahepatic metastasis. The ORR was 47.7%. Research data demonstrate that most of the adverse events are mild grade one or two adverse events, and there are no grade four or more serious adverse events. At present, the overall data is not yet mature, the mPFS and mOS have not yet reached, and the research is still in progress, which is worth looking forward to.

##### Panaprizumab

Panaprizumab is a PD-1 monoclonal antibody developed by a joint venture established by KANGFANG biomedical Co., Ltd. and China Institute of Biopharmaceuticals. It is characterized by complete removal of FC receptor and complement mediated function of Panaprizumab through FC mutation, and slow antigen binding and dissociation rate. These characteristics make it possible for Panaprizumab to become an anti-PD-1 drug with better clinical benefits. At present, in China, the new drug application of Panaprizumab for the treatment of relapsed or refractory (R/R) classical Hodgkin’s lymphoma (R/R CHL) has been accepted by Center for Drug Evaluation (CDE) in May 2020 (Song et al., 2019; Mislang et al., 2020).and published in the International Symposium on gastrointestinal cancer (ASCOGI) in 2021. As of November 2020, the confirmed ORR, DCR, mPFS, 6-month PFS, and 6-month OS were 31.0, 82.8, 63.2 and 93.2%, respectively. The incidence of grade 3 and above TRAE associated with Panaprizumab or Anlotinib was 19.4%, and the incidence of serious adverse events associated with Panaprizumab or Anlotinib was 6.5%. Studies suggest that the combination of Panaprizumab and Anlotinib is safe and tolerable, as the first-line treatment for patients with advanced HCC (Kotasek et al., 2019; Shan et al., 2021), showing encouraging antitumor activity. At the same time, the results support the exploration of a phase III clinical trial (NCT04344158) for the first-line treatment of advanced HCC with higher doses of Anlotinib combined with Panaprizumab, and Panaprizumab combined with Anlotinib (10 mg, continuous for two weeks, withdrawal for one week).

#### PD-L1 inhibitors

PD-L1 inhibitors mainly include Atezolizumab, Avelumab and Durvalumab. There are few clinical studies on PD-L1 inhibitors in the treatment of advanced HCC.

##### Durvalumab

Durvalumab is a humanized monoclonal antibody (IgG1 K type) against programmed death receptor ligand 1 (PD-L1) expressed in Chinese hamster ovary cells (CHO). In 2017, a phase I-II clinical study on Durvalumab in the treatment of advanced HCC patients who failed to receive sorafenib (Wainberg et al., 2017) included 40 patients with advanced HCC. After treatment with Durvalumab (10 mg/kg), the results showed that ORR was 10%, DCR was 33.3%, mOS was 13.2 months, and the incidence of grade 3–4 adverse reactions was 20%. The effect of single drug treatment was good. At present, there are more and more clinical studies, and the clinical study of combined immunotherapy in lung cancer has a clear curative effect, and the clinical study in PLC is under study.

##### Atezolizumab

Atezolizumab is a monoclonal antibody that can bind to PD-L1 and block its interaction with PD-1 and B7.1 receptor. These include activation of anti-tumor immune response without inducing antibody dependent cytotoxicity. In the syngeneic mouse tumor model, blocking PD-L1 activity resulted in decreased tumor growth. The phase 1b clinical study was reported at the ESMO meeting in 2019 (Stein et al., 2018). The ORR of 59 patients with advanced HCC treated with Atezolizumab as the first-line treatment was 17%. Compared with Atezolizumab combined with bevacizumab, the mPFS was 3.4 and 5.6 months, respectively. However, more and larger phase III clinical studies are needed to further confirm its efficacy and safety.

#### CTLA-4 blocker

On 25 March 2011, the United States Food and Drug Administration (FDA) approved the use of CTLA-4 monoclonal antibody (Ipilimumab) in the treatment of advanced melanoma, which has become a major breakthrough in the field of immunotherapy (Trinh and Hwu, 2012). Although the drug research stage has a good survival rate, but combined with a variety of related adverse reactions, it is criticized. In 2015, the US FDA granted the qualification for Tremelimumab, a monoclonal antibody against CTLA-4 inhibitor of AstraZeneca, for the treatment of mesothelioma. Tremelimumab is a human IgG2 monoclonal antibody against CTLA-4. Binding with CTLA-4 can prevent it from binding with B7 ligand, thus inhibiting the decline of T cell activity mediated by B7-CTLA-4. It can stimulate the immune system and anti-tumor effect. CTLA-4 inhibitors can recognize and eliminate tumor cells by enhancing the activity of antigen-presenting cells and T lymphocytes. A phase II clinical study (Sangro et al., 2013) (NCT01008358) included 20 patients with advanced PLC. After Tremelimumab treatment, ORR was 17.6%, DCR was 76.4%, mPFS was 6.48 months, mOS was 8.2 months, and viral load decreased. At the same time, some studies have shown that CTLA-4 inhibitors can bring OS, PFS, DCR benefits to advanced HCC. At the same time, studies have revealed that Tremelimumab has anti hepatitis virus effect. It is believed that in the future, with the continuous exploration of relevant phase III clinical studies, the application prospect of this drug in the treatment of advanced HCC will be expansive, which may bring longer survival hope to more patients with advanced PLC.

## Double immunity

The effect of single drug immunotherapy is limited in patients with advanced HCC. Combined immunotherapy has become a research hotspot in the treatment of advanced HCC. Studies have shown that (Hellmann et al., 2016) the combination of ICIs with different mechanisms of action could improve the response rate and anti-tumor effect of patients. The combination of CTLA-4 and PD-1/PD-L1 monoclonal antibody inhibitor would raise the tumor response rate and generate the synergistic effect. In 2017, the phase I clinical study of Tremelimumab combined with Durvalumab in the treatment of patients with advanced HCC (Kanikarla Marie et al., 2021) illustrated that the ORR of Tremelimumab combined with Durvalumab was 8% (25 *vs.* 17%) higher than that of monotherapy, and the DCR of Tremelimumab combined with Durvalumab for 16 weeks was 67.5%. Subsequently, a randomized, open, multicenter phase III trial (Abou-Alfa et al., 2018) (NCT03298451) with more patient samples was conducted to study the efficacy of Durvalumab +/- Tremelimumab compared with sorafenib in the treatment of patients with advanced HCC. The trial expanded the sample number, and 1200 patients were expected to be recruited. The main end point was OS. In 2019, the American Society of Clinical Oncology (ASCO) reported the results of the Checkmate-040 update study (He et al., 2020). In the Checkmate- 040 multi cohort study, 148 patients with advanced HCC who failed to receive sorafenib were included in this study. The results showed that the total ORR was 31% and the *duration of remission*.

(DOR) was 4.6–30.5 months, OS also achieved good results. At the same time, the meeting also reported the results of combined treatment of Nivolumab, Ipilimumab and Cabozantinib. The results indicated that the total ORR of 35 patients was 29%, the OS of 15 months was 70%, and the 3/4 grade of trail was 71%. Of course, a variety of clinical studies on the combination of ICIs in the treatment of patients with advanced HCC are in progress, which is worth to expect.

## Immunosuppressant combined with molecular targeted drugs

Molecular targeted drugs for PLC have been proved to have anti -angiogenesis effect, which can affect the immune response of PLC (Tian et al., 2017). In 2018, the ASCO reported a phase IB clinical study of Atezolizumab combined with bevacizumab in the first-line treatment of advanced HCC (Stein et al., 2018). The results showed that 21 cases had an evaluable ORR of 62%, and the effect was obvious. The results of the international multicenter phase III clinical study (Finn et al., 2018) (imbrave150) released at the ESMO meeting in 2019 further confirmed that Atezolizumab combined with bevacizumab is a new first-line regimen for the treatment of advanced HCC superior to sorafenib. Atezolizumab combined with bevacizumab significantly improved the overall survival of advanced HCC. The 6-month mOS rate of the combination group was 85%, the mOS did not reach, and the mPFS was 6.8 months; The 6-month mOS rate of sorafenib group was 72%, the mOS was 13.2 months, the mPFS was 4.3 months, the ORR of combined group was 27%, and the ORR of sorafenib group was 12%. Studies have shown that (Ikeda et al., 2018) Lenvatinib combined with PD-1/PD-L1 monoclonal antibody has a synergistic effect, which can block PD-1 immune escape pathway, and also inhibit monocytes to differentiate into TAM related to immune escape. At the same time, Lenvatinib would also inhibit the growth of tumor cells and TAM by competitively inhibiting the combination of VEGF and VEGFR. A phase IB clinical study was reported at the ASCO meeting in 2019 (Kudo et al., 2021). The results showed that the ORR of Avelumab combined with axitinib in the first-line treatment of 22 patients with advanced HCC was 31.8%, and the mOS was 12.7 months, while the ORR of single drug Avelumab in the treatment of advanced HCC was 13.6%. ASCO conference in 2020 reported a phase IB clinical study Keynote-524 (Finn et al., 2020). The study showed that the total ORR of Lenvatinib combined with pembrolizumab in the treatment of patients with advanced HCC was 46%, and the mPFS and mOS were 9.3 and 22 months respectively. The main safety aspects were proteinuria and elevated alanine aminotransferase. The results showed that the efficacy and safety of Lenvatinib combined with ICIs were better than monotherapy. It is worth further exploring in the treatment of PLC by combination drug therapy. In 2020 ASCO gastrointestinal conference, a phase IB clinical study of first-line treatment of advanced HCC with Nivolumab combined with Lenvatinib was reported (Kudo et al., 2020). The preliminary results indicated that mPFS was 7.39 months and ORR was 76.7%. The ASCO meeting in 2020 also reported a phase Ib/II clinical study on the first-line treatment of patients with advanced HCC with Panaprizumab (PD-1) combined with Anlotinib (Jiao S. C. et al., 2020). A total of 22 patients were included. The results showed that the total DCR was 84%, the median OS was not reached, and the 6-month OS was 91.6%. The data were satisfactory. At the same time, a phase III clinical study of Lenvatinib combined with pembrolizumab is in progress. In the second-line treatment clinical research, the phase I clinical trial of Camrelizumab combined with Apatinib in the treatment of patients with advanced HCC who failed to be treated by sorafenib (Mei et al., 2021). The experimental group was treated with Camrelizumab combined with Apatinib, Camrelizumab 200 mg, once every 2 weeks, and the control group was treated with single drug Apatinib. The clinical data proved the clinical benefits of ORR, DCR, mPFS, etc. There are 6 related wall reports in 2020 ESMO annual meeting, all of which are phase I/II studies. The drugs are selected in different scenario, including first-line and second-line schemes, but the safety and effectiveness are validated in all. The reported drug regimens include: Apatinib combined with Camrelizumab, Lenvatinib combined with CS1003, Sintilimab combined with Anlotinib, etc (Xu et al., 2019; Xu et al., 2020; Chen et al., 2021). Cabozantinib plus atezolizumab might be a treatment option for select patients with advanced HCC (NCT03755791), mPFS was 6·8 m (99% CI 5·6–8.3) in the combination treatment group *versus* 4·2 m (2·8–7.0) in the sorafenib group (hazard ratio [HR] 0·63, 99% CI 0·44–0.91, *p* = 0·0012). mOS (interim analysis) was 15·4 m (96% CI 13·7–17·7) in the combination treatment group *versus* 15·5 m (12·1-not estimable) in the sorafenib group (HR 0·90, 96% CI 0·69–1.18; *p* = 0·44) (Kelley et al., 2022).

## Immunotherapy combined with chemotherapy

In the past, the overall therapeutic effect of chemotherapy for PLC is not satisfactory. Studies have confirmed that chemotherapy boost the exposure of tumor cells to antigens, which is conducive to the immune effect of ICIs and enhance anti-tumor efficacy. Studies have shown that domestic ICIs combined with classical chemotherapy sometimes bring a surprising curative effect. A multi-center phase II study on the first-line treatment of advanced HCC with Camrelizumab combined with FOLFOX4/GEMOX, 34 cases were evaluable, the confirmed ORR was 26.5%, and the mDOR has not yet reached, DCR was 79.4%; mPFS was 5.5 m. At the same time, the tolerance and related adverse reactions of patients were acceptable (Qin et al., 2019), in other words, combined immunochemotherapeutic is an optional choice for advanced PLC patients. In addition, a randomized, open, national multicenter phase II/III clinical trial of first-line treatment of advanced HCC with Treprizumab combined with bevacizumab analogue and FOLFOX4 regimen is expected.

## Immunoneoadjuvant therapy

In 2020, the ASCO annual meeting published a research report on neoadjuvant therapy. A total of 30 patients with resectable PLC were enrolled in the study. Three cycles of bevacizumab combined with Ipilimumab or bevacizumab monotherapy were served for HCC before operation. After surgical resection, the pathological complete remission rate was 24% (3 cases in combination group, 2 cases in monotherapy group), and the main pathological remission rate was 16% (necrotic effect, 2 cases in combination group, 1 case in monotherapy group) (Yau et al., 2019a). In 2021, ASCO-GI reported a study on neoadjuvant treatment of borderline resectable or locally advanced HCC with Cabotinib combined with navulizumab (Yau et al., 2020), and the results showed that 12/15 achieved marginal negative resection, 5/12 of the patients who had been operated successfully achieved complete pathological response.

## Adjuvant therapy

With the first-line, second-line and neoadjuvant treatment of PLC have achieved gratifying outcome, the application of target therapy in adjuvant treatment of PLC is also constantly in exploration. For example, in June 2020, a multi-center study explored the adjuvant effect of iodine Metoximab after radical resection of PLC. 156 HCC patients with CD147 expression were included in the study. Patients in the treatment group were injected with iodine (Li et al., 2020) rituximab *via* hepatic artery once 4–6 weeks after operation. Results of the 5-year RFs time in the treatment group was significantly increased compared with that in the blank control group (43.4%: 21.7%, *p* = 0.0031), suggesting that the adjuvant regimen can improve the prognosis of patients. The study suggests that patients with PLC should consider on more accurate treatment protocols according to the classification of biomarker subgroup. At the 2020 ASCO annual meeting, the mid-term analysis results of a multi-center, prospective cohort study (Lance study) on the adjuvant therapy of Lenvatinib combined with transcatheter arterial chemoembolization (TACE) (Gao et al., 2010)for patients with HCC at high risk of recurrence after operation were released. A total of 90 patients with high risk of recurrence after radical surgery were enrolled in this study (macrovascular or bile duct invasion/tumor rupture or invasion of adjacent organs/grade II microvascular invasion with any of the following: tumor number ≥3, maximum tumor diameter ≥ 8cm, unclear tumor margin or incomplete capsule). The results showed that the median disease-free survival (DFS) time of patients in the combination group was significantly longer than that in the TACE group (12.0 *vs*. 8.0 m, *p* < 0.05); HR = 0.5,*p* = 0.0359) (Kudo et al., 2019). These results verify that the adjuvant therapy of Lenvatinib combined with TACE is not only effective and safe, but also prolong the PFS of HCC patients with high recurrence risk.

## Transformation therapy

The real-world study of PD-1 combined with TKIs for the treatment of PLC reported by Professor Sun Huichuan in 2020CSCO? in China shows that PD-1 combined with TKIs could be applied into the treatment of PLC. In 60 patients with advanced unresectable PLC, 11 patients were converted to resectable after receiving PD-1 combined with TKIs. When the data was published, 9 patients completed the operation, 5 of them achieved pathological complete remission, and the estimated survival time was more than 1 year (Cheng et al., 2019). 2020 ESMO-Asia reported a prospective, non-randomized, open cohort study (Zhu et al., 2021) of Professor Lu Shichun’s team in Beijing 301 Hospital: the study of HCC transformation therapy of TKIs combined with PD-1 inhibitor in the treatment of vascular invasion. As of 20 May 2020, a total of 70 patients were screened and 39 patients were included, of which 35 patients received combined treatment and 30 patients with PVTT, there were 2 cases of venous tumor thrombus and 3 cases of both. The criteria of successful transformation: 1. Child Pugh score <7; 2、ECOG PS≤1; 3. There was no extrahepatic lesion; 4. The hepatic vascular structure was intact and FLR was sufficient. The results showed that the median follow-up time was 7.2 months, no recurrence rate was 60% 6 months after operation, OS and RFs were still not to the end point, and the conversion and resection rate was 30.3%. At the same time, Professor Huang Cheng of Zhongshan Hospital in Shanghai, China, has also made some explorations in this field. As of December 2020, 23 unresectable HCC patients have been enrolled. After TKI combined with PD-1 antibody treatment, the tumor has been transformed and resected.

## Immunotherapy combined with local therapy

Previous studies (Marincola et al., 2000) have shown that radiotherapy is closely related to immunotherapy. Radiotherapy can affect the immune microenvironment of PLC cells and stimulate the production of some inflammatory cytokines (Presicce et al., 2009). Immunotherapy can also increase the sensitivity of radiotherapy; At the same time, radiotherapy also has the influence on immunosuppression. Radiotherapy can promote the body to produce immune response, induce the tumor cells to produce immunogenic death, and activate T lymphocytes. It recognizes and kill tumor cells in and out of the radiotherapy field, leading to the occurrence of the so-called “distant effect” after radiotherapy of malignant tumors. Therefore, in theory, radiotherapy combined with immunotherapy would obtain better curative effect. Since the Pacific study confirmed that immunotherapy consolidation therapy can be used as the standard treatment for unresectable non-small cell lung cancer after concurrent chemoradiotherapy, the survival benefit of this kind of tumor patients has been improved to a new height. So, what are the clinical results of immunotherapy combined with radiotherapy in the treatment of advanced HCC? PD-L1 expression level was monitored after local radiotherapy in the experiment. The results showed that radiotherapy combined with PD-L1 group had a significant inhibitory effect on tumor growth. The 7-week survival rates of combined treatment group, radiotherapy group and PD-L1 group were 90, 30 and 0%, respectively.

In the aspect of immunotherapy combined with microwave ablation of PLC, there is also preliminary theoretical evidence that ablation of PLC can produce a large number of inflammatory factors, a variety of immunogenic mediators and chemokines to play an anti-tumor role (Zhang et al., 2017). At the same time, ablation of PLC can increase the expression of HSP70, which may be one of the important reasons for the enhancement of anti-tumor immunity (Nikfarjam et al., 2005). Studies have shown that CTLA-4 monoclonal antibody combined with thermal ablation in the treatment of advanced PLC can significantly reduce the viral load of patients with hepatitis BLC, down regulate Treg cells in TME, increase the infiltration of CD8+T cells in tumor site, and increase the survival rate of patients with advanced PLC, the 6-month and 12-month DFS rates were 57.1 and 33.1%, respectively (Duffy et al., 2017; Kudo and Masatoshi, 2017).

The most important advantage of TACE in the treatment of PLC is to effectively treat PLC and avoid liver injury. After TACE, malignant tumor cells are lysed and necrotic, which can produce a large number of highly immunogenic cell components, thus initiating the related immune response (Zavadil et al., 2019). Tremelimumab combined with TACE (NCT01853618) is considered as an adjuvant therapy for advanced HCC. About 26% of the preliminary results show that it is partially effective. A phase III clinical study (NCT03778957) explored the efficacy of TACE combined with bevacizumab and Durvalumab in patients with locally advanced HCC. At the same time, another study (NCT0281754) evaluated the efficacy of Durvalumab combined with Tremelimumab (TACE/RAF/cryoablation) in the treatment of advanced HCC. These clinical studies are in progress. In general, the large-scale clinical research of ICIs combined with local therapy for PLC has been or is in progress, which is worth looking forward to.

In recent years, local arterial infusion chemotherapy (HAIC) for PLC has achieved satisfactory results, and has become a “new star” in the research of local treatment of PLC (Verhoef et al., 2008). In 2020, Professor Shi Ming retrospectively analyzed the efficacy and safety of Lenvatinib + Toripalimab + HAIC in 157 patients with advanced PLC (He et al., 2021b). The results showed that the triple therapy group showed longer PFS (11.1 *vs*. 5.1 m, *p* < 0.001); Longer OS (less than: 11 m, *p* < 0.001); And higher ORR (RECIST: 59.2%: 9.3%, *p* < 0.001); mRECIST:67.6%∶16.3%, *p* <0.001); The higher DCR rate (RECIST or mRECIST: 90.1 *vs*. 72.1%, *p* = 0.005) (He et al., 2021a) indicated that HAIC combined with Lenvastinib and Treprizumab could significantly improve the efficacy of treatment on patients with advanced PLC. These studies preliminarily show that HAIC has a remarkable effect in the treatment of advanced PLC.

## Challenge and thinking of immunotherapy for liver cancer

ICIs have been widely used in the clinical treatment of advanced HCC, and achieved excellent results. It has changed the new pattern of systemic treatment of HCC. At present, the combination therapy of immunosuppressive agents for HCC is emerging in an endless stream. With the positive results of clinical studies and the accelerated approval of FDA, the combination therapy will become the mainstream of the treatment of HCC in the future. However, with the progress of clinical practice, there are many problems and challenges to be solved in immunotherapy of HCC, such as the requirement of exploration more effective combination modes, optimization of the existing drug treatment sequence, development of new drug and cell combination schemes, the choice of biomarkers, the challenges of drug economy and safety, etc.

### Effective biomarkers for predicting efficacy

Although immunosuppressive agents for HCC have shown very encouraging efficacy, they also face the challenge of drug resistance. Therefore, it is very important to find effective biomarkers in the evaluation. PD-L1 is a common biomarker of malignant tumor in clinic, but the clinical guiding value of PD-L1 is different for different malignant tumors. Professor Pinato’s study showed that the positive expression rate of PD-L1 in (Pinato et al., 2019) PLC cells was less than 10%, while the correlation between the positive expression of PD-L1 and the efficacy of ICIs was not found in Keynote-224 and Checkmate-040 studies (El-Khoueiry et al., 2017; Zhu et al., 2018). Whether the expression of PD-L1 is related to the efficacy of PD-1/PD-L1 monoclonal antibody in the treatment of advanced HCC is still uncertain. Some clinical studies (Carbone et al., 2018; Klempner et al., 2020) have shown that TMB is an independent predictor for evaluating the therapeutic effect of ICI for a variety of tumors, and TMB is positively correlated with the efficacy of immunotherapy (Klempner et al., 2020). The results of the clinical trial Keynote ⁃ 158 make the tumor mutation load the second tumor associated diagnostic marker approved for clinical application (Marabelle et al., 2020). However, some studies have also shown that compared with other malignant tumors, TMB is the second tumor associated diagnostic marker approved for clinical application, the expression level of tumor mutation load in HCC is not significant, and its predictive value of curative effect is limited (Yarchoan et al., 2017). Therefore, the predictive effect of TMB on the curative effect and the determination of the cut-off value still need to be further explored. In addition, mismatch repair defects and microsatellite instability only occur in 2–3% of PLC patients, and their application value in PLC is very limited. In 2019, ESMO reported the predictive value of neutrophil to lymphocyte ratio (NLR) and platelet lymphocyte ratio (PLR) in the treatment of PLC with Nivolumab. The results show that the degree of lymphocyte infiltration in TME is closely related to the heterogeneity of HCC.

There is a correlation regions with high tumor heterogeneity have higher degree of immune infiltration and better response to immunotherapy (Losic et al., 2020). Harding et al. (Harding et al., 2019). Showed that patients with CTNNB1 mutation had poor response to PD-1 inhibitors, which may indicate the predictive role of CTNNB1 mutation in curative effect. Therefore, the clinical value of PD-1, TMB, microsatellite instability and other commonly used biomarkers in the treatment of PLC is limited, and more research is needed to find more effective biomarkers.

### How to improve the combination therapy strategy

In addition, the data of local treatment combined with systemic treatment is still insufficient. All kinds of phase I/II combination therapy show promising efficacy, but there is still a lack of confirmed results of phase III study. Immunotherapy combined with targeted therapy or double immunotherapy has a high ORR, which also provides the possibility of transformation therapy for unresectable or borderline resectable PLC (Tarantino and Curigliano, 2020). However, at present, there is no clinical recommendation of the most effective combination mode. With the continuous improvement of evidence-based medicine, systematic treatment may participate in the whole process of advanced PLC treatment. The specific combination therapy, interventional therapy timing and dose course still need to be further standardized and refined.

### Real-world research status of ICIs

At present, many large-scale clinical studies of ICIs in the treatment of malignant tumors have achieved satisfactory results (Mazdak et al., 2021), but in the real world, ICIs in the treatment of HCC do not have such a convincing effect (Shintaro et al., 2008). Randomized controlled trials (RCTs) are generally accepted in the medical community to evaluate the safety and effectiveness of drugs, but the inclusion and exclusion criteria of RCTs are too strict. The results of the study are not completely consistent with the real-world research, and it is also a difficult problem to constantly select the dominant population for refinement.

### Progress of immunization

The research of ICI has brought a breakthrough in the field of tumor research. Immunosuppresses can effectively inhibit tumor growth and progress of disease e.g.HCC. However, immuno hyperprogress is a tough topic to face. Champiat et al. (Champiat et al., 2017) proposed that the definition of the super progress is that the time to treatment failure (TTF) is less than 2 months, the tumor load increases by more than 50%, and the progress pace (PP) increases more than twice as much as the progress. The incidence of immune hyperevolution is about 4–30%, which may be related to the types of tumor. The pathogenesis of immune hyperprogress is not clear. Some studies have reported the potential clinical and biological predictors of hyperprogress (Demets, 2013). The possible predictors are over 65 years old, two or more metastasis sites, gender, low expression of PD-L1, etc. Of course, the diagnosis criteria of the super progress caused by immunotherapy needs the involvement of histopathology.

### How to transform “cold tumor” into “hot tumor"

HCC is a special high immune type cancer, most of the patients belong to the characteristics of “cold tumor immune cycle”. Cold tumor is the tumor with no or little immune cell infiltration around it (Galon and Bruni, 2019). Cold tumor with poor prognosis, is often the most difficult tumor to treat while “hot tumor” is the opposite. The transformation of cold tumor into thermal tumor is usually realized through the combination therapy. Most of the cold tumors can be transformed into thermotumor by direct infusion of activated immune cells *in vitro* through the adoptive cell therapy, and the immune effect ability can be enhanced. It can also be transformed into thermotumor by combining radiotherapy and chemotherapy and local treatment (Gabriele et al.). At present, the understanding of “cold and hot tumor” is not comprehensive, and the research on the transformation of “cold tumor” is still a big challenge in immunotherapy.

### Security challenges

With the wide application of immunotherapy and the acceptance of immunotherapy drugs in medical insurance, the price of immunotherapy drugs has decreased significantly, and the incidence of related adverse reactions has further increased, such as immune myocarditis, immune pneumonia, immune hepatitis, enteritis, etc. How to reduce the incidence of immune related adverse reactions would be a giant challenge in the near future (Lee et al., 2021).

## Summary and prospect

At present, the treatment of PLC has entered the era of immune 3.0, especially the application of ICIs has become the most promising drug for the treatment of PLC. Although the effect of single immunotherapy is accountable, the combined immunotherapy shows us the new hope in the treatment of PLC. In a number of studies, we have seen that the combined immunotherapy has achieved the consequence of 1 + 1 > 2. For example, the combination of Atezolizumab and bevacizumab has achieved good results in both ORR and PFS (Wallin et al., 2016). In the future, we will continue to explore a variety of combination models based on immunotherapy, such as the combination of immunity and immunity, immunity combined with radiotherapy, immunity combined with ablation, immunity combined with chemotherapy and targeted drugs, immunity combined with intervention, etc. Meanwhile, we also need to think about how to combine treatment for patients with different types and stages, which is a raising problem we are facing. In the future, the drug resistance of immunotherapy is bound to affect the curative effect of PLC treatment. To study the drug resistance mechanism of PLC and the corresponding new drugs needs our continuous efforts and innovation, and further clinical research is required to provide evidence-based medicine to choose the optimal scheme for the individualized treatment of PLC patients.
